# Ankle Swelling in Patients With Type 1 Neurofibromatosis: A Report of Two Cases With Rare Presentation of Common Genodermatosis

**DOI:** 10.7759/cureus.28938

**Published:** 2022-09-08

**Authors:** Irene N Thomas, Amutha Balasundaram, Joseph Jenson James, Nithya Priyadharshini Shanmugam

**Affiliations:** 1 Department of Dermatology, Sri Sathya Sai Medical College and Research Institute, Ammapettai, IND; 2 Department of Dermatology, Government Medical College, Namakkal, IND

**Keywords:** cafe-au-lait macules, neurofibromatosis type 1, skeletal deformity, diffuse neurofibroma, solitary ankle swelling

## Abstract

Neurofibromatosis type 1 is characterized by multiple cutaneous neurofibromas of varying sizes along with skeletal, neurologic, and ophthalmic features. Solitary swellings in neurofibromatosis type 1 are not commonly encountered except in the form of plexiform neurofibromas. We report two cases with neurofibromatosis type 1 presenting with solitary swelling in the ankles which were proven to be the diffuse type of neurofibroma, radiologically and histopathologically. Diffuse type neurofibroma presenting as ankle swelling in type 1 neurofibromatosis has not been reported before.

## Introduction

Neurofibromatosis type 1 or von Recklinghausen disease is an autosomal dominant genodermatosis presenting with multiple cafe-au-lait macules, single or multiple neurofibromas, axillary and inguinal freckling, Lisch nodules, and skeletal and central nervous system abnormalities [1]. Cutaneous neurofibromas in neurofibromatosis type 1 are tumors of the skin that may be sessile or pedunculated. They are soft in consistency and usually increase in number as the affected person gets older. In these patients, cutaneous neurofibromas are numerous ranging in size from a few millimeters to several centimeters, usually distributed over the course of the peripheral nerves [2]. Solitary neurofibromas usually present as plexiform neurofibromas. Uncommonly, neurofibromas can occur solitarily as diffuse neurofibroma over the head and neck areas [3]. We report two cases of histopathologically proven diffuse neurofibromas presenting as ankle swellings in type 1 neurofibromatosis. Solitary ankle swelling presenting as diffuse neurofibroma in type 1 neurofibromatosis has not been reported so far.

## Case presentation

Case 1

An 18-year-old female presented in the department of dermatology in a teaching hospital in Chennai with the chief complaint of diffuse swelling of the right ankle for the past eight years. The swelling was insidious in onset, gradually increased in size, and was not associated with pain. There was a history of multiple pigmented lesions over the trunk since birth. There was no history of similar swellings elsewhere in the body and no history of central nervous system involvement such as seizures, visual disturbances, or hearing impairment. There was no positive family history, she was born of non-consanguineous parents and her developmental milestones were normal.

**Figure 1:**
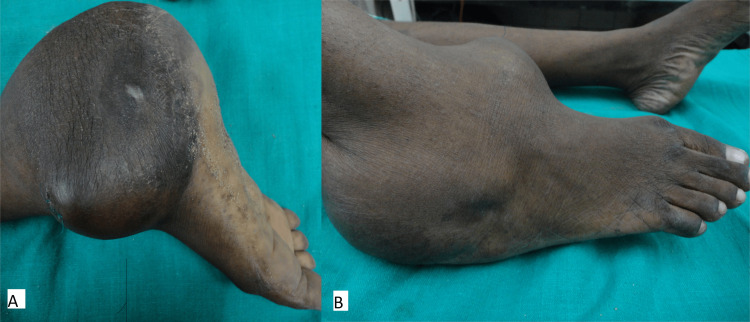
Medial view of ankle swelling (A) and lateral view of ankle swelling (B).

On examination, a single irregular swelling of size 15 cm × 11 cm was present encircling the right ankle. The swelling was soft in consistency and not tender on palpation (Figures 1A, 1B). It did not have the bag of worms feel and peripheral nerves were not thickened.

**Figure 2:**
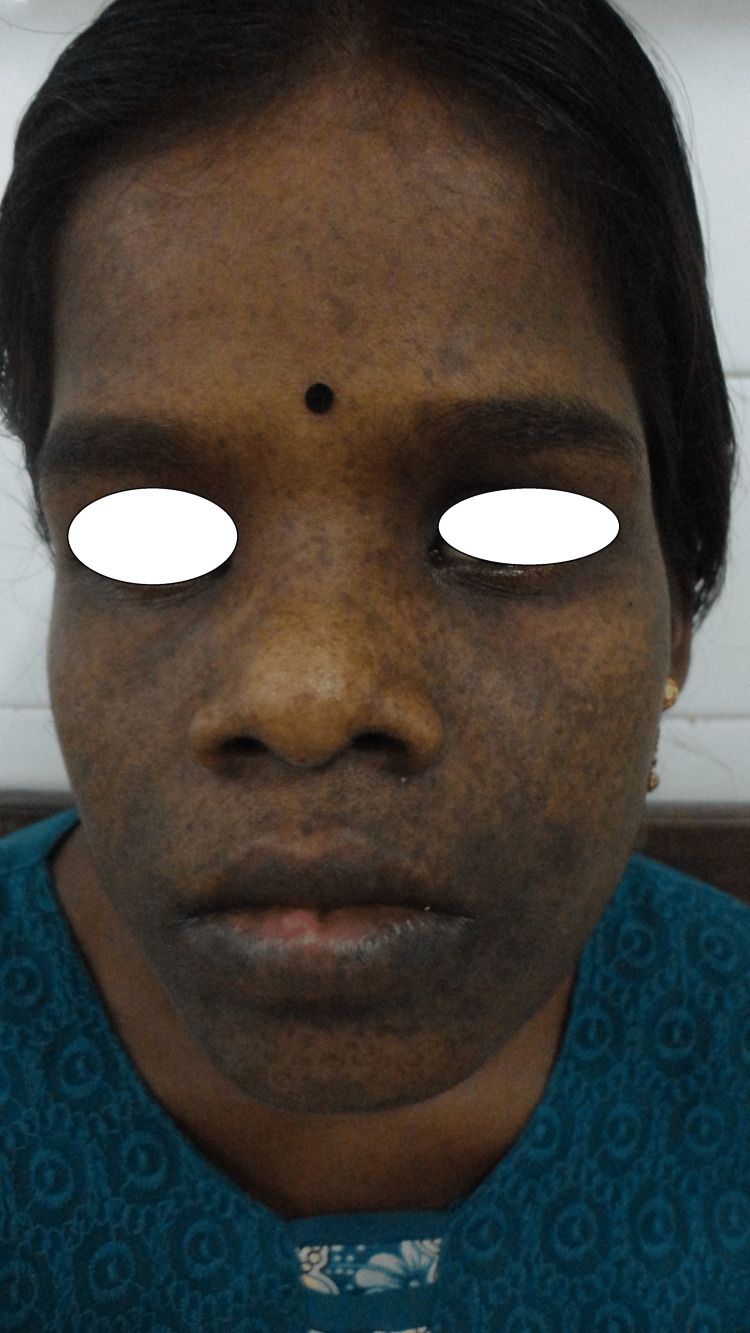
Pigmented macules present bilaterally over the cheeks and lips.

**Figure 3:**
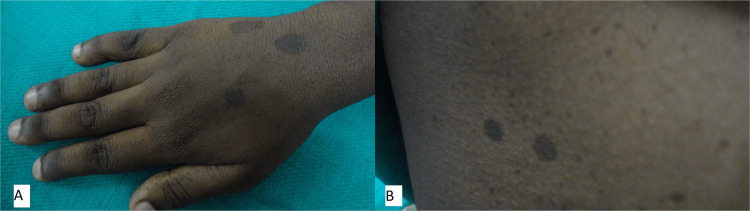
Cafe-au-lait macules on the dorsum of right hand (A) and cafe-au-lait macules on the trunk (B).

Numerous pigmented macules were present bilaterally over the cheeks and lips (Figure 2) along with multiple café-au-lait macules of varying diameters from 0.5 cm to 11 cm over the trunk, and hand (Figures 3A, 3B). Taking into account the extensive freckling of the face the possibility of xeroderma pigmentosum was considered in the differential diagnosis. However, the patient did not have a positive family history, nor did she have photosensitivity.

**Figure 4:**
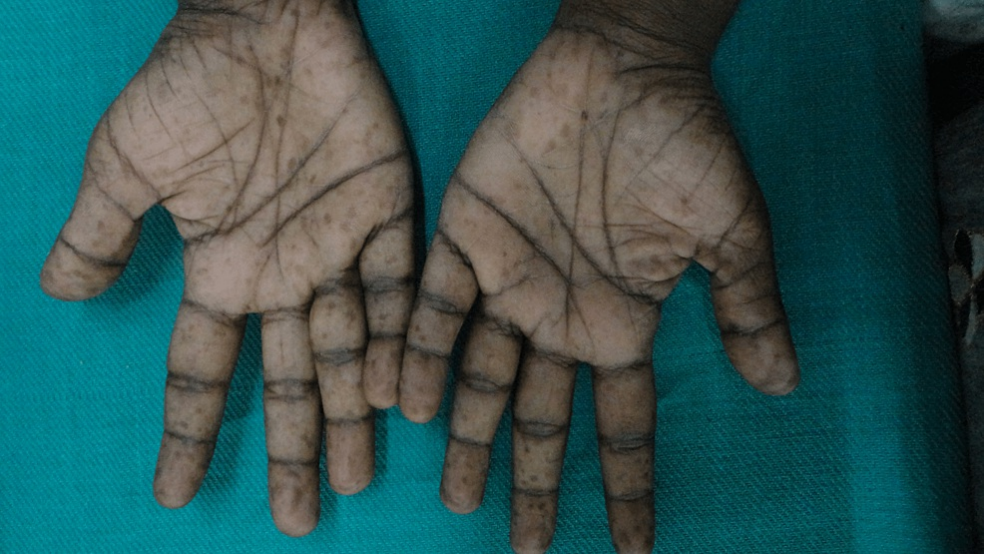
Freckles in both palms - Case 1.

Multiple hyperpigmented macules noted to be as freckles were seen in both palms (Figure 4). The patient also had Lisch nodules in the left eye and kyphoscoliosis. Clinical examination of other systems was normal and vital signs were stable. Clinical diagnosis of neurofibromatosis type 1 was made as the patient fulfilled the Revised National Institute of Health consensus criteria (Table 1).

**Table 1:** Revised National Institute of Health consensus criteria 2021. The table is adapted from Legius et al. [4]. Sib: sibling; NF1: neurofibromatosis type 1; HPE: histopathology

S. no.	Criteria	Case 1	Case 2
1	Six or more café-au-lait macules over 5 mm in greatest diameter in prepubertal individuals and over 15 mm in greatest diameter in postpubertal individuals	Present	Present
2	Freckling in the axillary or inguinal region bilaterally	Present	Present
3	Two or more neurofibromas of any type or one plexiform neurofibroma	Single large diffuse neurofibroma around the ankle (proved with HPE)	Single diffuse neurofibroma around left medial malleolus (proved with HPE)
4	Optic pathway glioma	Absent	Absent
5	Two or more iris Lisch nodules identified by slit lamp examination or two or more choroidal abnormalities	Present	Present
6	A distinctive osseous lesion such as sphenoid dysplasia, anterolateral bowing of the tibia, or pseudarthrosis of a long bone	Present	Absent
7	A first-degree relative (parent, sib, or offspring) with NF1 as defined by the above criteria	Absent	Absent

**Figure 5:**
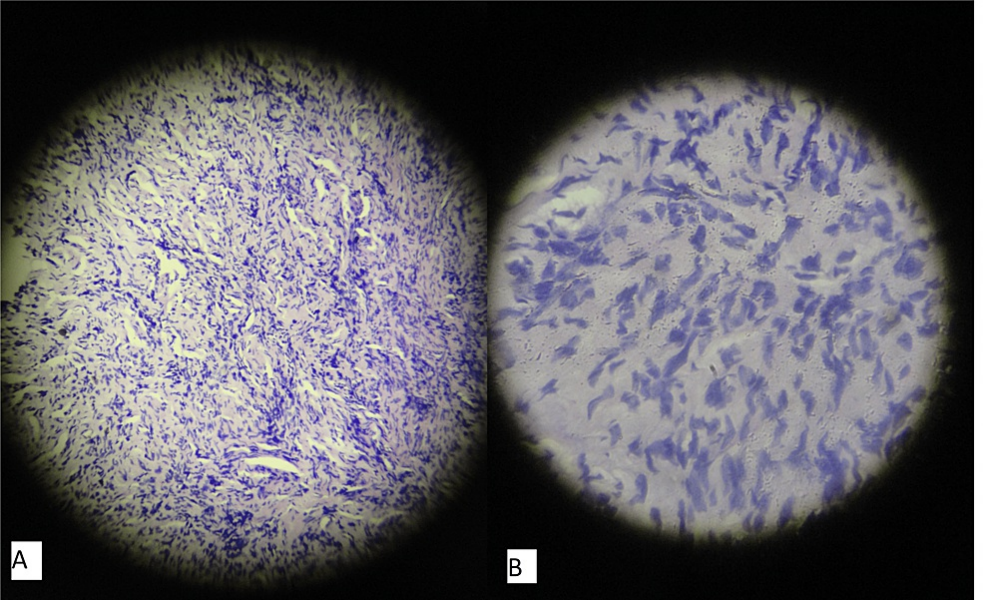
Low power view (10×) H&E stain of the biopsy specimen (A) and high power view (40×) H&E stain of the same (B). Dermis shows unencapsulated tumor composed of spindle cells with elongated, wavy nuclei regularly spaced among thin wavy collagenous strands merging into the adjacent fascia.

On investigations laboratory parameters were normal. A biopsy was taken from the swelling and histopathology revealed a normal epidermis, with the dermis showing an unencapsulated tumor composed of spindle cells with elongated, wavy nuclei regularly spaced among thin wavy collagenous strands merging into the adjacent fascia (Figures 5A, 5B).

**Figure 6:**
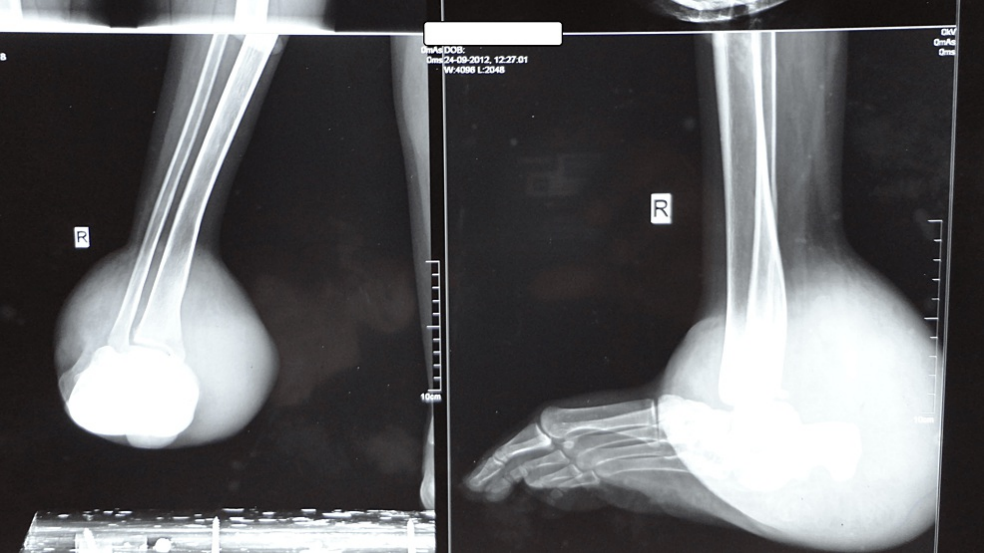
X-ray of the right ankle (oblique and lateral view) shows soft tissue shadows around the ankle joint with anterolateral bowing of the tibia.

**Figure 7:**
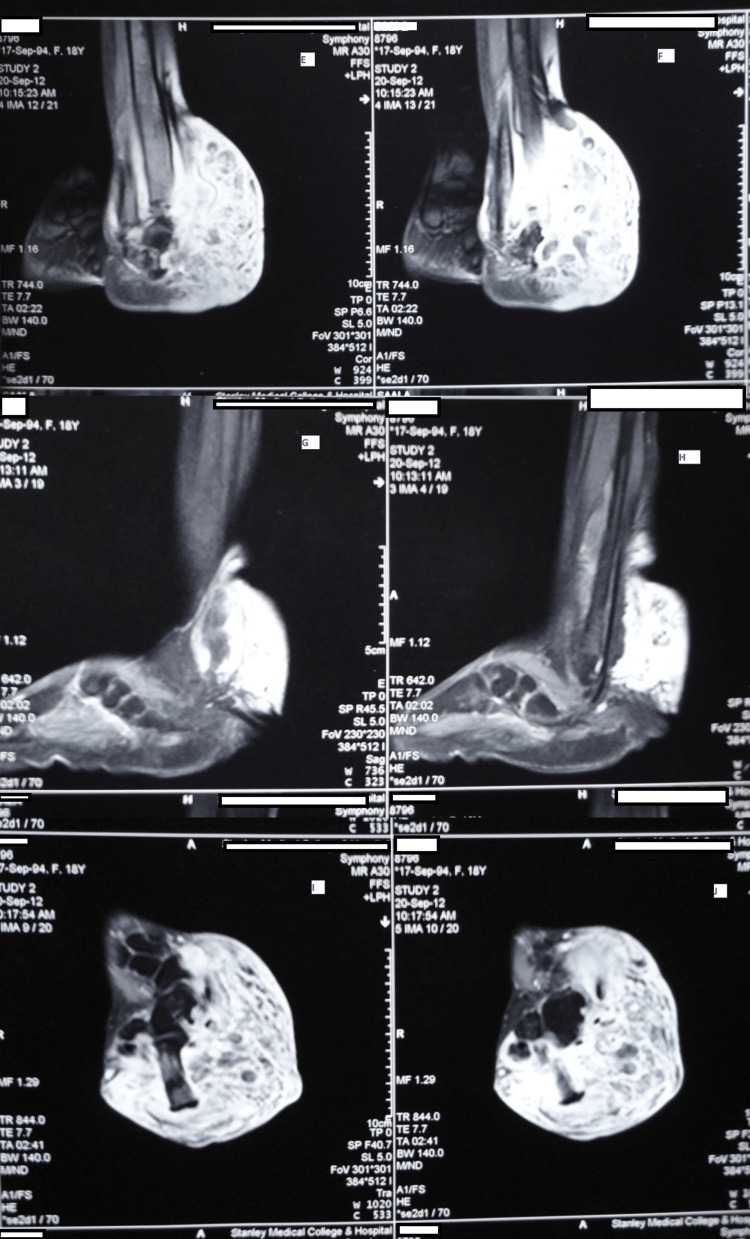
MRI of the right ankle swelling was described as T1 hypointense and T2 hyperintense lesion in the posterolateral aspect of the ankle.

**Figure 8:**
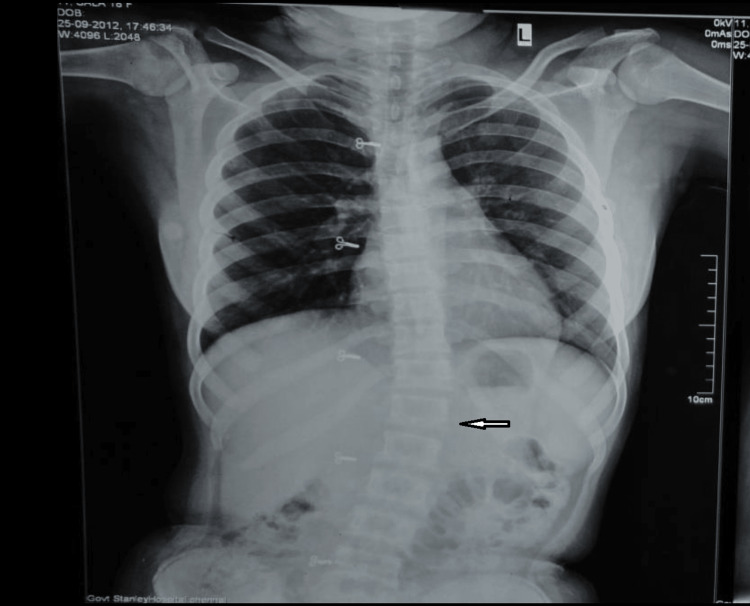
X-ray chest and spine (PA view) shows kyphoscoliosis at T10-L2 vertebral levels (arrow). PA: posteroanterior

Skiagram of the affected limb showed soft tissue shadows around the ankle joint with slight bowing of the tibia (Figure 6). MRI of the swelling of the right ankle was reported as T1 hypointense and T2 hyperintense lesion in the posterolateral aspect of the ankle extending from the subcutaneous plane crossing the interfascial plane with altered signal intensity noted in lower one-third of the tibia with minimal bony expansion (Figure 7). Spine x-ray confirmed kyphoscoliosis (Figure 8). The patient was referred to the plastic surgery department for excision of the neurofibroma and to the orthopedics department for correction and management of kyphoscoliosis.

Case 2

**Figure 9:**
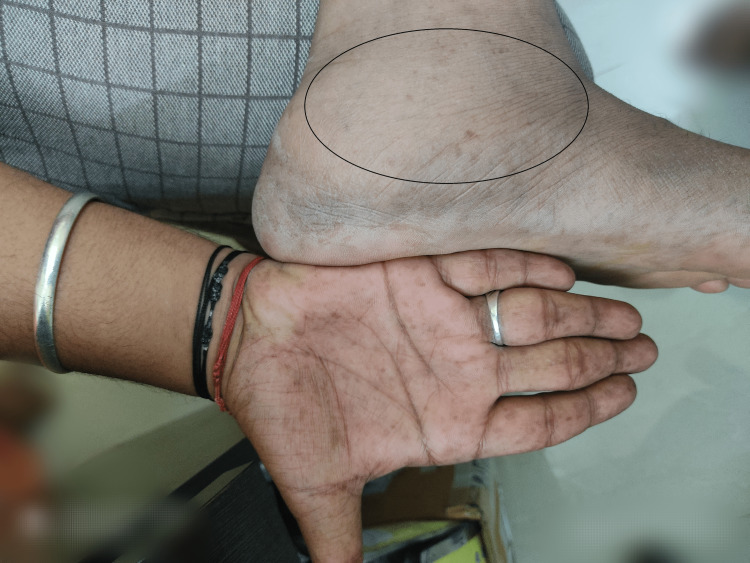
Subcutaneous swelling over the left ankle with palmar freckling (marked within circle).

A 24-year-old male presented in the dermatology department in a teaching hospital in Chennai with a swelling of the left ankle for five years which was insidious in onset and was gradually increasing in size (Figure 9). There was no family history suggestive of neurofibromatosis (NF) type 1 and developmental history was normal.

**Figure 10:**
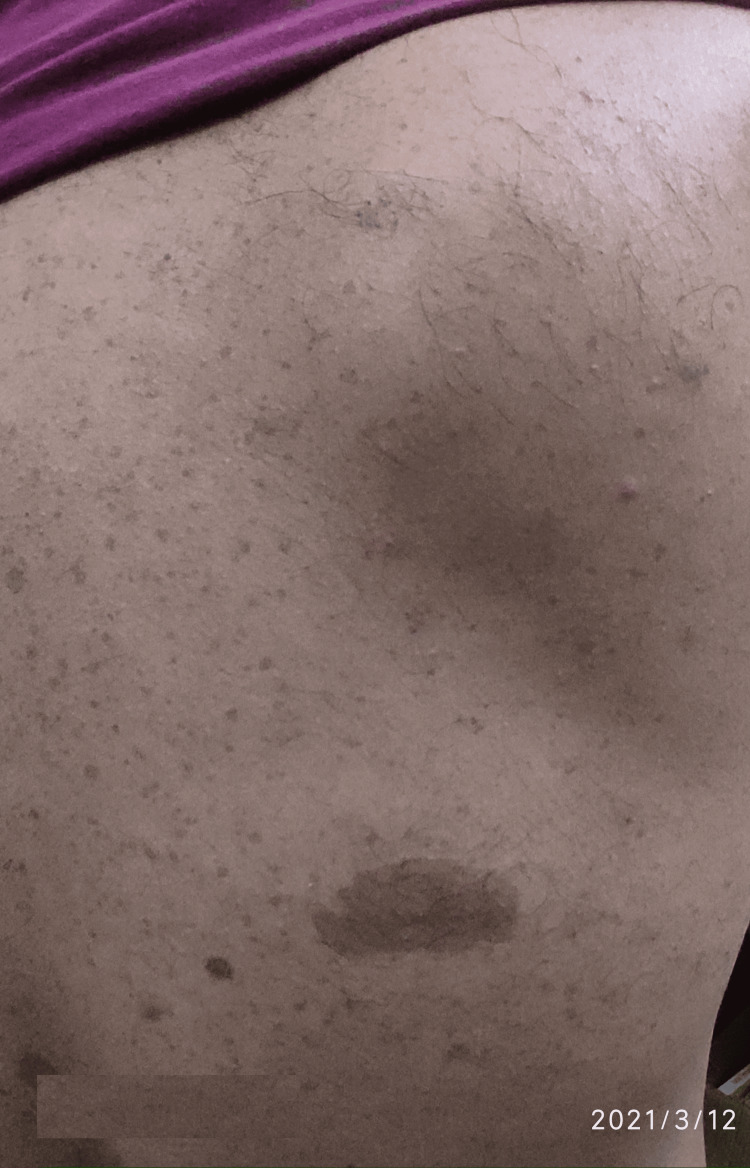
Multiple cafe-au-lait macules over the back.

**Figure 11:**
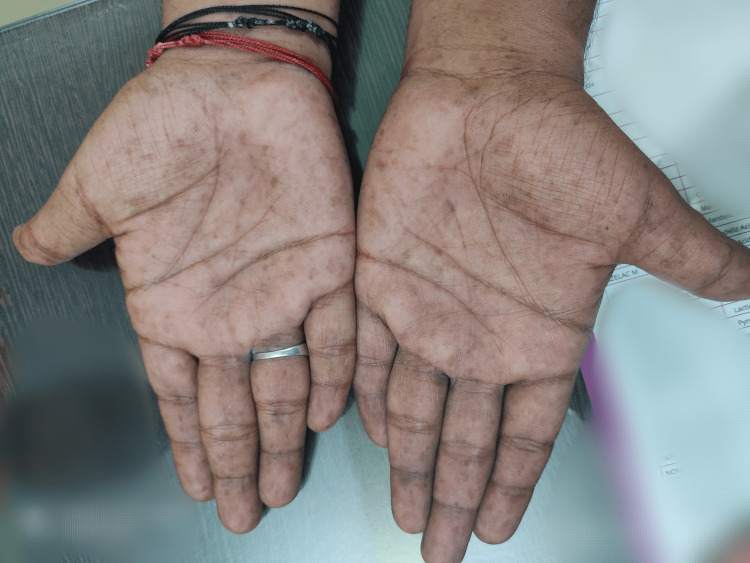
Freckling in both the palms - Case 2.

**Figure 12:**
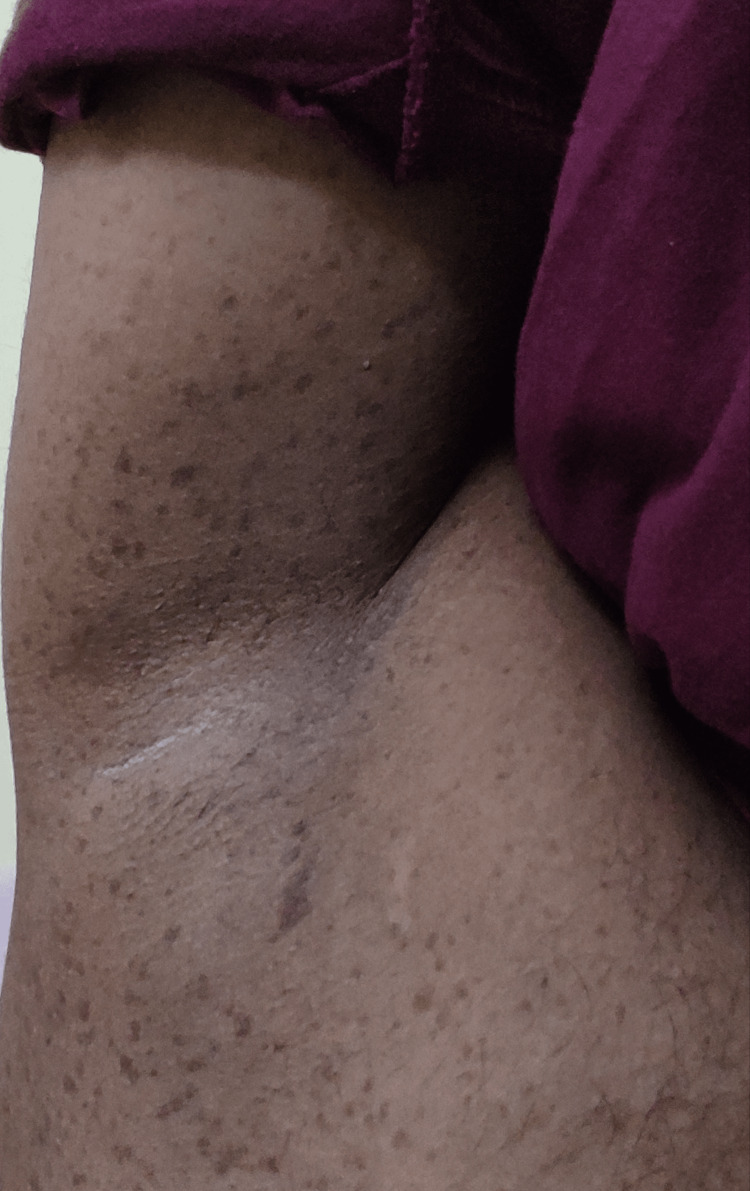
Freckling over the axilla - Case 2.

The general examination was normal and vital signs were stable. There was no neurological deficit. The patient had multiple café-au-lait macules of varying sizes ranging from 2 cm to 5 cm over the back and inguinal region (Figure 10). He also had freckles over the palms, axillae, and inguinal region distributed bilaterally (Figures 11, 12).

**Figure 13:**
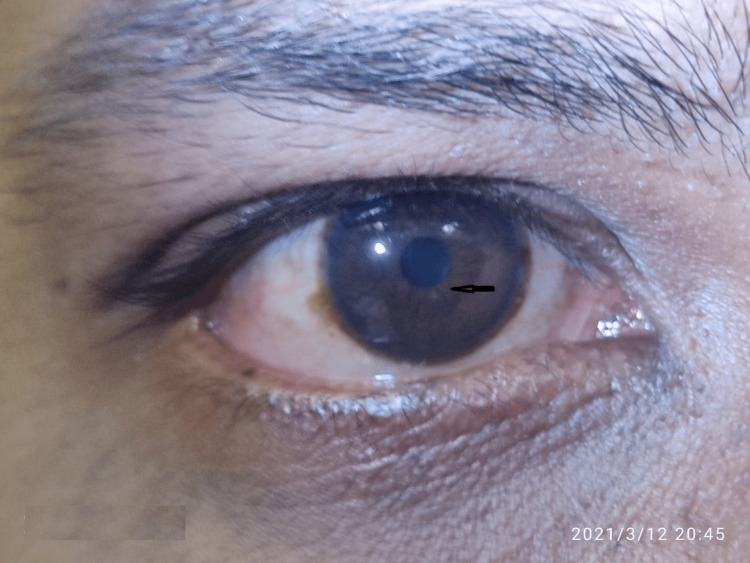
Arrow shows Lisch nodules below the iris at 6 o'clock position in the right eye.

Two Lisch nodules found in the right eye were confirmed by the ophthalmologist (Figure 13). This patient also fulfilled the revised diagnostic criteria for the diagnosis of neurofibromatosis type 1 (Table 1).

In this patient, an 11 cm × 7 cm subcutaneous swelling was also present over the medial aspect of the left ankle obscuring the medial malleolus. The swelling was uniformly soft without the irregular bag of worms consistency and was not tender. The peripheral nerves were not thickened or palpable.

**Figure 14:**
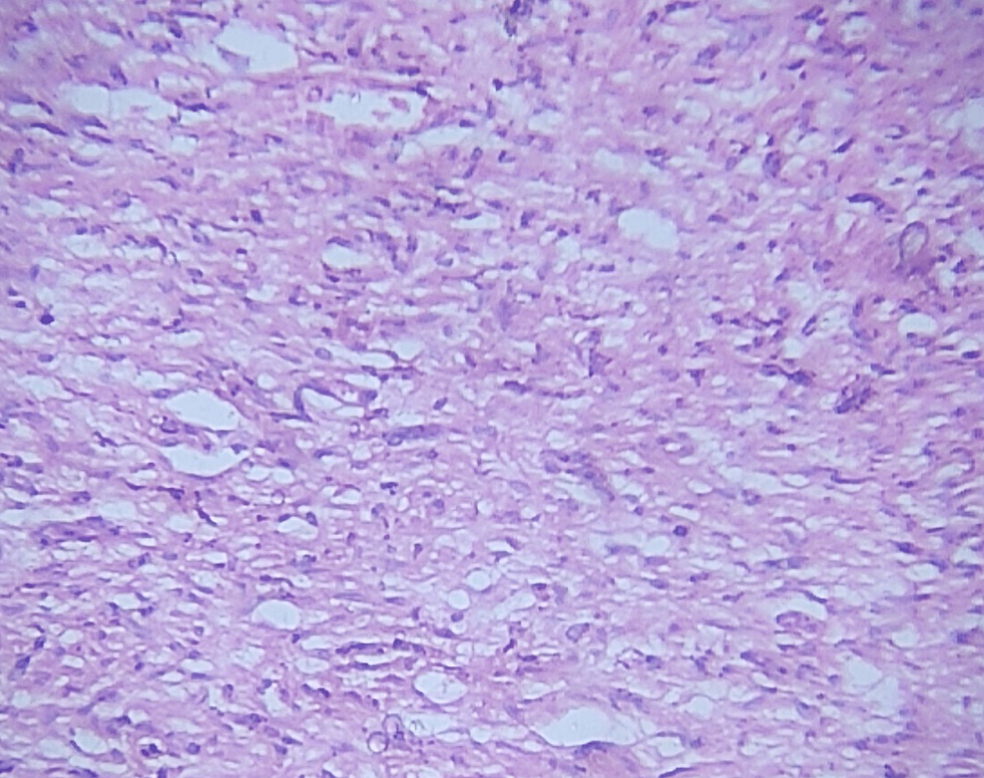
Low power (10×) H&E stain showing unencapsulated tumor of cells with oval or spindle-shaped nucleus.

A biopsy was done from the swelling that exhibited features of neurofibroma with the dermis showing an unencapsulated tumor of cells with an oval or spindle-shaped nucleus and scant, indefinite cytoplasm, merging imperceptibly to the surrounding tissue (Figure 14). A clinical diagnosis of solitary diffuse neurofibroma of the ankle was made which was confirmed histologically.

The diagnostic criteria for NF1 are met in an individual who does not have a parent diagnosed with NF 1 if two or more of the following are present: though both patients did not have a parental history of neurofibroma, they fulfilled three of the diagnostic criteria of the revised diagnostic criteria for NF 1 as seen in Table 1 - (1) café -au-lait macules of the required dimensions, (2) bilateral inguinal and axillary freckling, and (3) Lisch nodules.

## Discussion

Neurofibromatosis type 1, according to Riccardi's classification, is an autosomal dominant inherited syndrome manifested by developmental changes in the nervous system, bones, and skin. In type 1 neurofibromatosis, also called Von Recklinghausen’s disease, patients have multiple neurofibromas, cafe-au-lait spots, axillary freckling, and other skeletal and ophthalmic manifestations. Diagnostic criteria for NF1 have been defined taking into account these features [4]. Most of the patients present as classical cases with generalized manifestations.

Classical neurofibromas are benign tumors that present as cutaneous tumors of varying sizes, numbers, and morphology. They rarely occur before late childhood, but continue to develop throughout life. These tumors usually arise from the nerve sheaths of the peripheral nerves or cranial nerves varying in number from a few to hundreds. They can be invaginated, as if through a ring in the skin, by pressure with the finger, called the buttonhole sign. Plexiform neurofibromas present as large, irregular bag-like swellings with the typical bag of worms appearance and occur along the course of a nerve [5].

When neurofibromas present as solitary tumors they are usually seen as localized plexiform neurofibromas as stated in Table 1. Diffuse neurofibromas are a newly recognized entity [6]. Our report highlights the rare occurrence of solitary diffuse neurofibroma around the ankle joint in two patients with NF1 who did not have any other skin tumors.

Diffuse neurofibromas usually manifest as diffuse dermal plaque-like swellings in the head and neck regions resulting in enlargement of the affected tissue [7]. These are uncommon with less than 10% presenting as diffuse neurofibromas in neurofibromatosis type 1 [8]. Solitary neurofibromas presenting as the diffuse type are rarer and solitary diffuse neurofibroma in the axial skeleton presenting as ankle swelling have not yet been reported so far. Both our patients revealed moderately large diffuse swelling around the ankle joints which were soft in consistency and not tender. No peripheral nerve thickening and bag of worms appearance was noted ruling out plexiform neurofibromas.

On investigations, skeletal x-ray of the affected limb in both patients showed soft tissue shadows around the ankle joint with a slight curvature of the tibia in one of the patients. MRI of the ankle swelling revealed a T1++ hypointense to T2 hyperintense lesion in the posterolateral aspect of the ankle. These findings were similar to the MRI findings of diffuse neurofibromas reported in the literature [9]. In addition, altered signal intensity was noted in the lower third of the tibia along with minimal bone expansion in Case 1.

Histologically, diffuse neurofibromas are described to be unencapsulated spindle cell tumors with bland cytologic appearance, serpiginous nucleo-cytoplasmic contours, and poorly delimited boundaries with the surrounding dermis [10]. This was the histopathological picture seen in our patients. Also, in both of our patients, the diagnosis of diffuse cutaneous neurofibroma was confirmed histologically and radiologically.

Nerve sheath tumors such as neurofibromas and schwannomas are quite rare around the foot and ankle accounting for 5.4% and 3.9%, respectively, of all benign soft tissue tumors in this region [11]. Usually, these benign nerve sheath tumors are solitary and are not associated with NF 1.

Diffuse solitary neurofibromas can lead to complications such as limb hypertrophy and dysfunction. Unilateral hypertrophy in an extremity not only involves soft tissue hypertrophy but can also infiltrate the long bone cortex with narrowing of the medullary canal with cortical thickening [12]. In addition to functional consequences, unilateral hypertrophy of the extremities can cause cosmetic disfigurement.

Solitary neurofibromas not associated with NF 1 rarely become malignant, but when associated with the NF1 syndrome, they has the potential for malignant transformation. It has been reported that 12% of patients with neurofibromatosis type 1 will eventually develop malignant nerve sheath tumors from the neurofibromas of the oral cavity [13]. Solitary diffuse neurofibromas can also have the risk of undergoing malignant change, although the quantification of such risk has not been determined [14,15].

Unilateral enlargement of the ankle swelling causing cosmetic and functional deformity and the risk of potential malignant transformation led us to refer our patients to plastic surgery where the swellings were excised successfully with a good cosmetic and functional outcome.

## Conclusions

This report of two cases with the rare presentation of solitary diffuse neurofibromas around the ankle joint in NF1 emphasizes the awareness that clinicians should have in encountering unusual manifestations of common genodermatoses.

Arriving at the right but rare diagnosis among the spectrum of differentials in unilateral ankle swelling involves good history taking and an astute clinical examination. In our patients, pigmentary lesions such as café-au-lait macules, axillary and palmar freckling, and Lisch nodules helped us to arrive at the diagnosis of NF1 without the typical multiple cutaneous neurofibromas. Biopsy of the solitary ankle swelling established the diagnosis of diffuse neurofibroma in addition to the radiological findings. We recommend that this unusual variant of NF1 presenting as solitary diffuse neurofibroma around the ankle joint be included among the various clinical manifestations of neurofibromatosis type 1.
